# Study Comparing Topical Ivermectin Versus Topical Permethrin in the Treatment of Scabies

**DOI:** 10.7759/cureus.48746

**Published:** 2023-11-13

**Authors:** Afshan Saeed, Shahan Tariq, Majid Iqbal, Hamza Ismaeel, Asher Mashhood, Musarrat H Raza, Muhammad Ammar Hamid

**Affiliations:** 1 Dermatology, Pak Emirates Military Hospital Rawalpindi, Rawalpindi, PAK; 2 Dermatology, National University of Medical Sciences, Rawalpindi, PAK; 3 General Internal Medicine/Gastroenterology, Services Hospital Lahore, Lahore, PAK; 4 Pediatrics, Rawalpindi Medical University, Rawalpindi, PAK; 5 Internal Medicine, University of South Dakota Sanford School of Medicine, Sioux Falls, USA

**Keywords:** ivermectin, sarcoptes scabiei var. hominis, topical agent, permethrin, scabies treatment protocol

## Abstract

Introduction

Scabies is a highly contagious skin disease caused by an ectoparasite mite called *Sarcoptes scabiei*. Ivermectin and permethrin have been commonly used for the treatment of scabies. However, topical ivermectin has been compared to other treatment modalities to a lesser extent.

Objective

This study aimed to compare the efficacy of topical ivermectin versus topical permethrin in the treatment of uncomplicated scabies.

Methods

354 patients with scabies attending the dermatology outpatient department of Pak Emirates Military Hospital Rawalpindi were enrolled. Patients were divided into two groups randomly. The first group and their family contacts received 1% ivermectin lotion whereas the other received 5% permethrin lotion. Patients were evaluated at the end of the second and the fourth week.

Results

At the end of the second week, initial follow-up showed that 97 out of 159 patients (61.0%) in the ivermectin 1% group, and 107 out of 159 patients (67.3%) in the permethrin 5% group had achieved clinical cure (P=0.24). On the final follow-up at the end of Week 4, the cure rate amounted to 85.5% (136 of 159 patients) in the ivermectin group and 89.9% (143 of 159 patients) in the permethrin group. Differences among both groups remained statistically insignificant (P=0.23).

Conclusions

The use of ivermectin 1% versus permethrin 5% as topical therapy showed almost identical results for the treatment of uncomplicated scabies. Side effects were minimal and there were no significant differences observed in patients with regard to compliance among both the groups.

## Introduction

Parasitic infectious infestations such as scabies are a growing problem and already a cause of global concern [1]. Although scabies is not unique to specific populations/races or genders, it is indigenous in communities of the developing world, especially those with limited access to clean water, low hygiene levels, overcrowded areas such as hostels, nursing homes, orphanages, and prisons, not to mention the tropics [2,3]. The parasite mite, namely, *Sarcoptes scabiei var. hominis*, affects over 100 million people each year worldwide and leads to notable impairment in quality of life [1,4]. The disease can even occur and spread in asymptomatic individuals [5]. Scabies also has a significant causal association with impetigo, which may result in the progression of skin and soft tissue infections [6]. So far, permethrin is considered the most effective topical therapy for the treatment of scabies [7]. Ivermectin, a time-tested anti-parasitic drug, has been used to treat a number of various endoparasites as well as ectoparasites for the past 100 years [8]. Similarly, the efficacy of ivermectin has been proven in many studies using both oral and topical formulations [9-11]. However, only a few studies/trials have been conducted to date [12]. Moreover, comparisons regarding topical ivermectin have been infrequent, and mostly it has been studied as an oral therapy for the treatment of scabies [13-15]. Therefore, this randomized control trial was organized to help advance and reinforce the ever-evolving literature regarding the treatment effectiveness between topical treatment modalities.

## Materials and methods

We conducted a study at the outpatient clinic of the Department of Dermatology, Pak Emirates Military Hospital Rawalpindi. This single-blinded, randomized controlled, parallel-group clinical trial was carried out from September 1, 2020, to May 28, 2021. The sample size was calculated using Raosoft and it comprised of a total of 354 participants.

**Figure 1:**
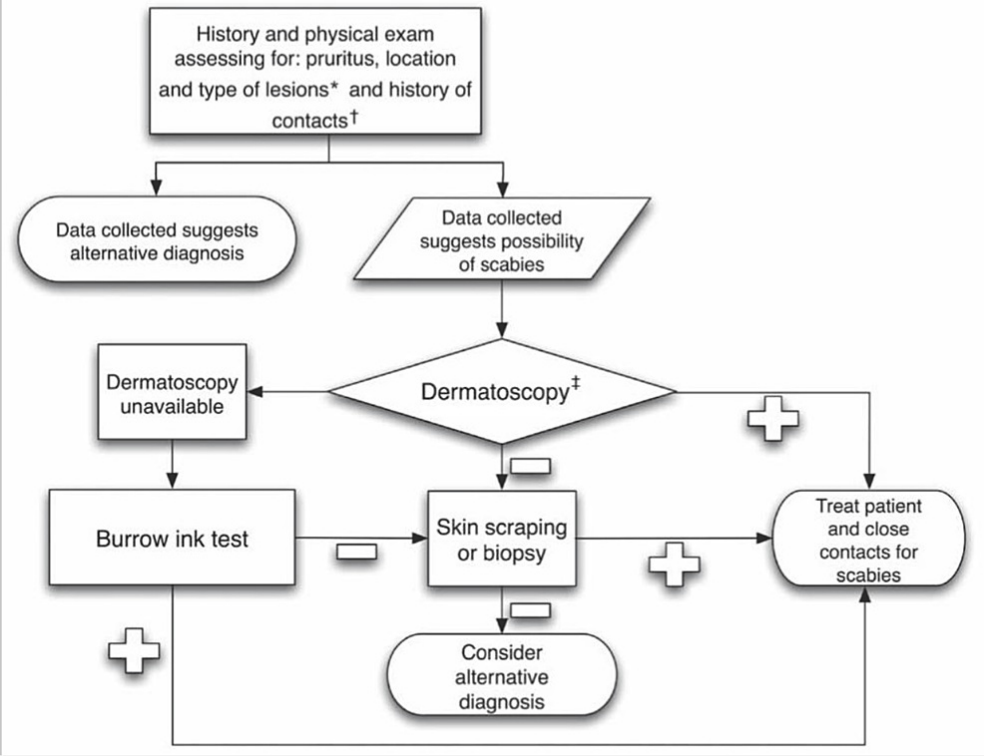
Proposed clinical algorithm for diagnosing scabies *Primary lesions are typically located in the finger webs, on the flexor surfaces of the wrists, on the elbows, in the axillae, on the buttocks and genitalia, and on the breasts. †Close contact may include family members and sexual partners. Healthcare workers with a history of caring for patients with scabies should be considered at risk. ‡Use of a hand-held dermatoscope requires training to recognize the typical ‘jet with condensation trail’ pattern and hence was performed by senior residents or consultants [17]

This cohort of 354 eligible patients was randomly selected, following due authorization from the ethical committee. Prior to their enrollment, patients were physically examined in detail. A thorough history of their infestations was obtained along with any recent antibiotic treatment and other pertinent information regarding their household/occupational contacts was recorded. For the purpose of demographic comparison as well as randomization later, a record of all the patients' ages, height, weight as well as gender, was made. Diagnosis of scabies infestation in patients was made by either the presence of a burrow site and classical scabetic lesions such as vesicles, nodules, or papules at common locations of infestation, nocturnal pruritus and similar symptoms reported by the household/close contacts of the patients. Direct observation of the larvae, mites, or ruminants such as eggs or fecal matter under a light microscope confirmed the diagnosis [16]. Patients had to meet suitable diagnostic criteria to be enrolled in the trial (Figure 1) [17]. Those who met the criteria above were randomly assigned to two groups as mentioned below.

Before the commencement of the trial, patients received thorough and comprehensive information regarding the study, and subsequently, informed written consent was obtained. Information encompassing demographic variables such as age, gender, body weight, and the duration of the complaint was documented. These variables were used for the process of randomization, which was implemented using a computer-generated random list to allocate participants into their respective groups. The group assignments were securely enclosed within sealed envelopes and unveiled only upon the enrollment of each patient.

In total, 354 patients were enrolled when the trial began. Of these, 36 study subjects were lost to follow-up and thus excluded. Those remaining comprised 235 males and 83 females; a total of 318 patients. Out of 159 patients in Group A, 126 were male and 33 were female, whereas Group B consisted of the other 159 participants, 109 male and 50 female. Patients in Group A received a lotion of propylene glycol containing 1% ivermectin and was instructed to apply it thoroughly to all the affected areas leaving it for at least eight hours before bathing once weekly. The dosage was calculated to be 400 micrograms/kg. Patients in Group B received 5% permethrin lotion and was advised to apply it all the way from the neck to the toes for a minimum of eight hours before shampooing on a weekly basis. The treatment was provided to both the patients and their household contacts. All patients were instructed to be generous in the application of their respective medications and to avoid the usage of any other topical therapies during this time period. Additionally, patients were also prescribed oral antihistamines (Zyrtec), a dose of 10 mg once or twice daily for the management of moderate to severe pruritus respectively.

A non-probability consecutive sampling technique for the inclusion of patients was used and subjects with a confirmed diagnosis of scabies from 15 years of age up to the age of 50 years were included, irrespective of gender; the mean age being 35.981 ± 6.14 years. The average duration of complaints was 4.293 ± 1.86 weeks in our entire sample. Group A had a mean weight of 77.012 ± 7.78 kg and a mean age of 38.295 ± 6.32 years, with an average complaint duration of 4.163 ± 1.93 weeks. Group B had an average weight of 77.314±8 .67 kg, a mean age of 37.334 ± 7.02 years, with an average complaint duration of 4.423 ± 1.79 weeks (Table 1). Certain exclusion criteria were implemented such as patients who had received any topical and/or systemic scabicidal treatment in the previous month, any topical or systemic antibiotic usage within the week preceding the study, history of seizures or developmental disabilities, status of immunological compromise, severe disease including Norwegian scabies, ongoing or recent secondary bacterial infection, allergy towards the treatment drugs or any constituent thereof, stage II hypertension or a blood pressure of less than 100/60 mmHg. Those women who were pregnant or lactating were excluded from the study as well as children and the elderly. 

**Table 1:** Baseline characteristics of the treatment groups

Group	Male	Female	Mean weight (kg)	Age (years)	Complaint duration (weeks)
Group A	126	33	77.012 ± 7.78	38.295 ± 6.32	4.163 ± 1.93
Group B	109	50	77.314 ± 8.67	37.334 ± 7.02	4.423 ± 1.79

This study was done when COVID-19 was at its peak and, therefore, the exclusion of patients below the age of 15 and above the age of 50 years was done as a precautionary measure to minimize the time they spent in a hospital setting, thereby, reducing the potential risks for these age groups. Children and the elderly were identified as more vulnerable populations. Including them in the study and then following them up for four weeks could have introduced additional risks. Therefore, the exclusion of these age groups was not only a safety measure to minimize the potential for virus transmission, but also to protect these vulnerable populations as well as their contacts. For example, guardians/parents who mostly accompany minor patients had a chance to be exposed to the virus unnecessarily on multiple occasions had they been included in the study, leading to unfortunate outcomes and further spread of the virus. Additionally, the exclusion of patients with comorbidities, especially in the elderly population, was done as a further safety precaution. Elderly individuals often have a higher prevalence of comorbid conditions and, hence, they may be on multiple medications. The possibility of interactions between ivermectin and permethrin with the treatment/maintenance therapies of elderly patients could not be ruled out completely and, thus, it could have potentially influenced the study. Moreover, it was essential to consider interactions between medications, which can be complex, altering their efficacy or safety profiles. By excluding individuals with comorbidities, the study aimed to minimize the influence of these interactions on the results and ensure a more controlled assessment of the treatment's effectiveness.

**Figure 2:**
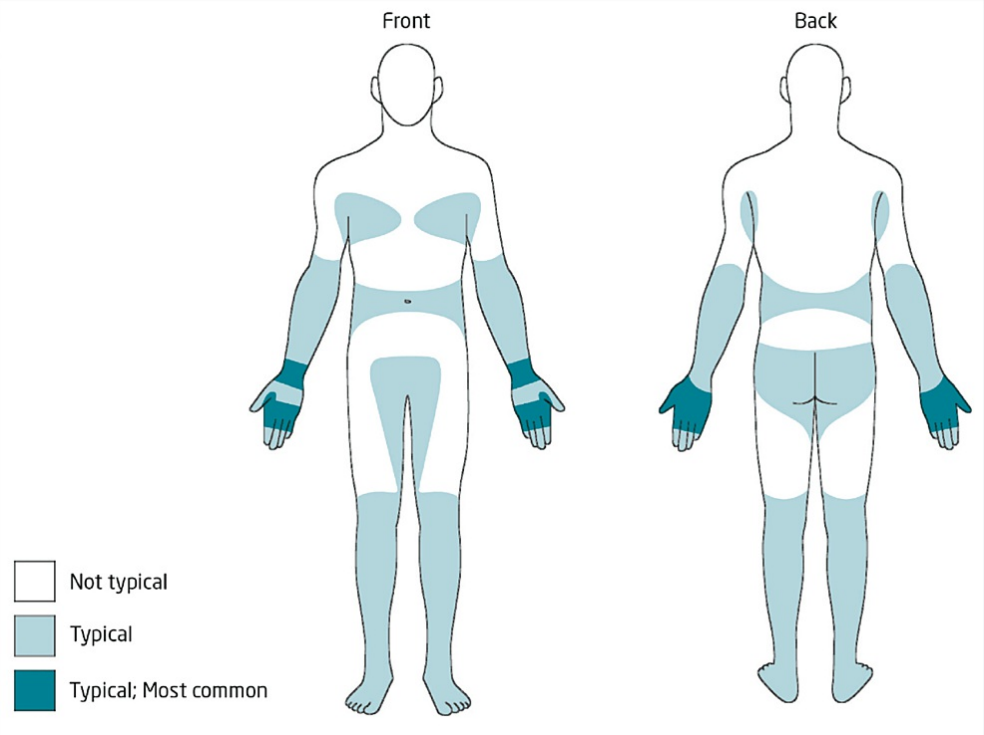
Body diagram of typical distribution of scabies lesions in adults In older children and adults, lesions are most commonly seen in an acral distribution, affecting the hands and feet. In the upper limb, involvement of skin distal to the mid-upper arm is considered typical. The most commonly affected areas are the volar wrists and the hands. On the hands, lesions are most commonly seen at the finger web spaces, knuckles and sides of fingers and hands. In the lower limb, involvement of skin distal to the mid-thigh is considered typical. The toes, including web spaces, are commonly affected. Other typical areas for lesions include the “belt” region, running circumferentially around the waist at the level of the umbilicus, the genital area and upper-inner thighs, buttocks and axillae. In females, the breasts, particularly the periareolar skin, are typical region [18].

All the patients were monitored during this trial and follow-up was conducted at the end of the second and fourth weeks. Patients were evaluated for compliance at every follow-up and inquired about household contacts during this period. The final evaluation of efficacy was done following the completion of the fourth week. Data pertaining to efficacy was recorded using a specifically tailored proforma whilst adhering to the pre-established operational criteria. At each follow-up, the lesion sites were marked on similar body diagram sheets for every patient (Figure 2), and a comparison was made with the lesions at the same locations that were visible in the pre-treatment photographs. Newer lesions were also evaluated either using dermoscopy or an optical light microscope after scrapping. Clinical examinations were conducted on all patients and history was taken regarding any side effects. Necessary counseling was done to keep the patients compliant and motivated to keep following up. All evaluations were based on criteria that have been defined previously [17]. The absence of any new scabies lesions along with the active healing of all the old lesions was defined as “cure”, regardless of the presence or absence of scabetic nodules. “Treatment failure” was depicted by the presence of new lesions and/or the failure of older lesions to resolve completely on follow-up at the end of the fourth week. In patients who had treatment failures or re-infestations, their respective treatment was continued and patients were assessed again at subsequent follow-ups. The trial period for all the patients including treatment failures ended at the completion of the fourth week. Treatment failures made up only a handful of patients though and were represented as uncured at the end of the study. Eventually, all of the patients had their scabies infestation treated successfully.

The χ² was used for the calculation of the mean difference between both groups. P value of <0.05 was considered statistically significant. Statistical analysis of efficacy and comparison was drawn using the Chi-square test. SPSS (version 16) was used for all the analyses.

## Results

**Figure 3:**
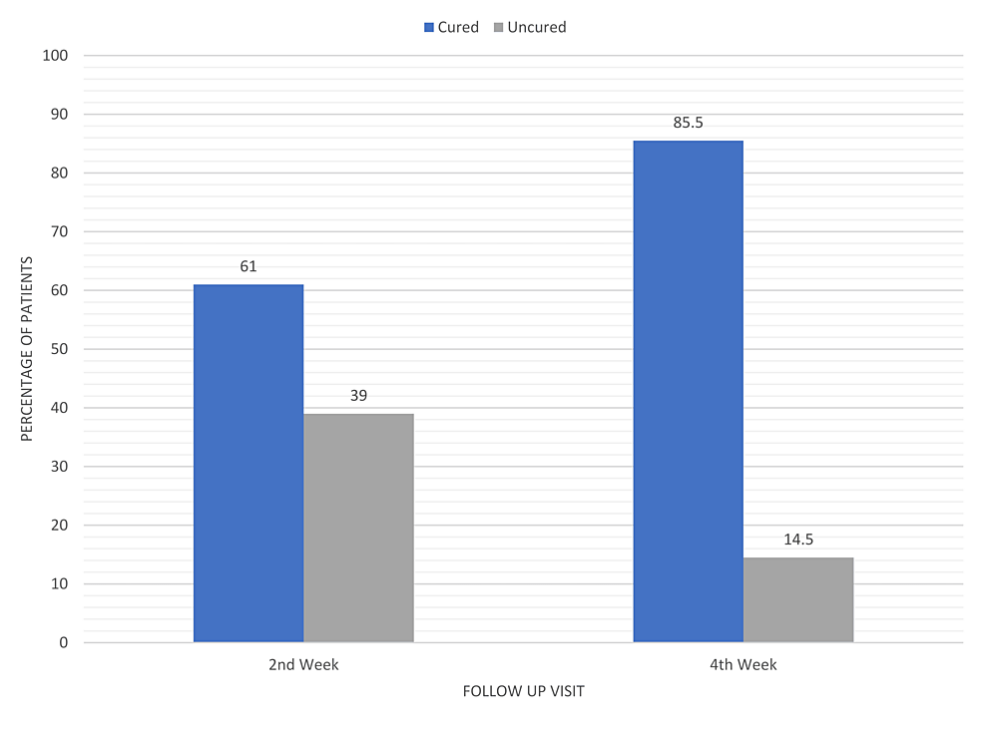
Clinical cure rate at subsequent visits in patients using topical ivermectin 1%

**Figure 4:**
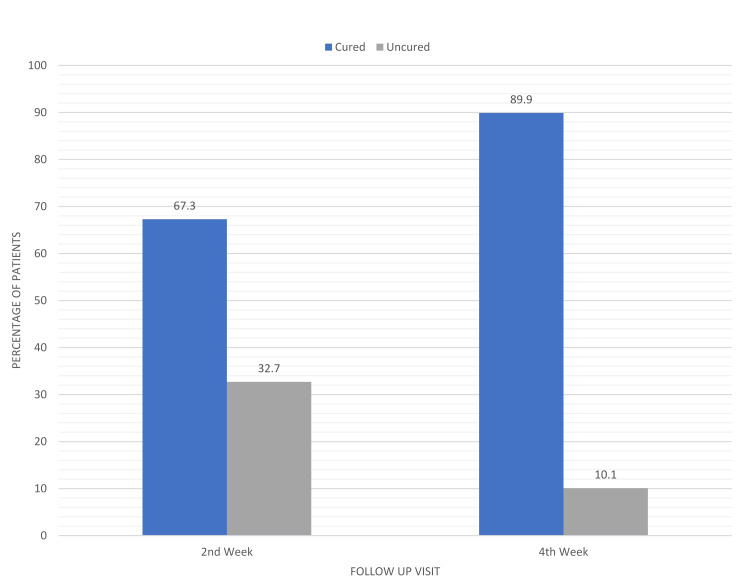
Clinical cure rate at subsequent visits in patients using topical permetherin 5%

**Figure 5:**
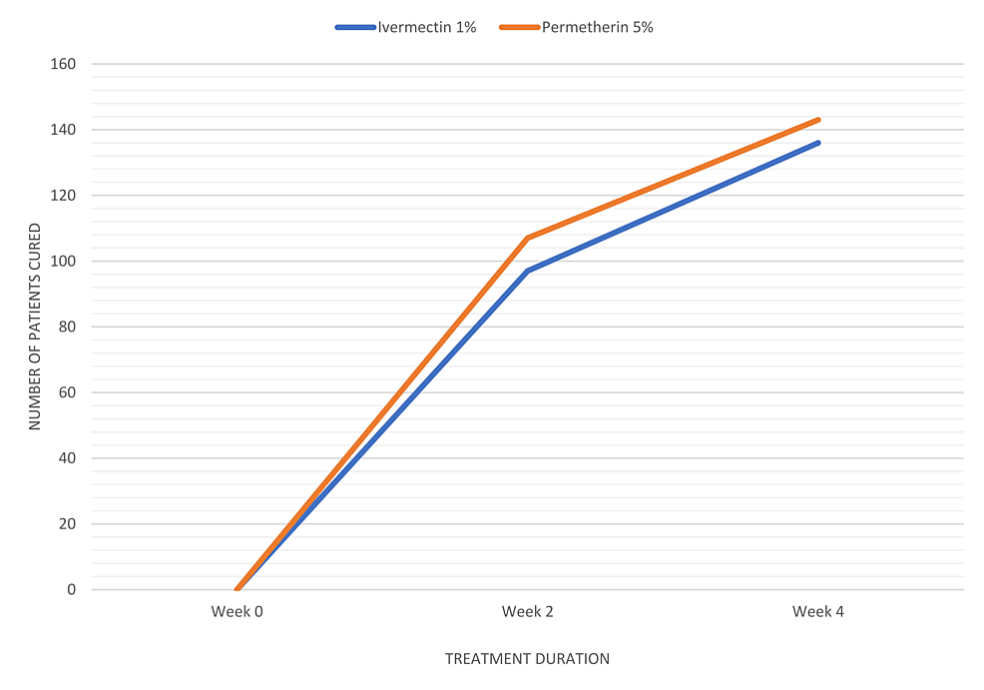
Number of patients cured after topical use of ivermectin 1% vs permetherin 5% Differences between treatment groups remained statistically insignificant (P>0.23)

The two groups did not show any significant differences with regard to age, gender, or the mean duration of complaint. Additionally, at the beginning of the study, there were no significantly notable variations between these groups with regard to the number of patients classified as having mild, moderate, or severe infestation. After a two-week follow-up, it was observed that 97 out of 159 patients (61.0%) in the ivermectin 1% group (Figure 3) and 107 out of 159 patients (67.3%) in the permethrin 5% group (Figure 4) had achieved clinical cure, although complete relief from pruritic symptoms was yet to be seen in some subjects. Neither statistical nor clinical significance of one treatment being superior to the other was observed (P=0.24). In patients who still had infestations (65 male and 49 female) after receiving treatment, the therapy was continued; a total of 114 individuals spread across both the medication groups. On the last follow-up at week 4, only 23 out of the 62 remaining patients in the ivermectin group continued to experience itching and skin lesions compared to the remaining 16 patients out of 52 in the permethrin group. Consequently, the overall cure rate amounted to 85.5% (136 of 159 patients) in the ivermectin group (Figure 3) and 89.9% (143 of 159 patients) in the permethrin group (Figure 4). Differences among them remained statistically insignificant (P>0.23) (Figure 5).

After the trial had ended, those patients who were considered as treatment failures in the study were retreated with the subsequent topical therapy they had received earlier respective to their treatment group. Only seven patients were noted to have resistant scabies, who were then treated further with the help of a combination of 1% lindane solution and permethrin 5%, which ultimately cured their infestation in the following one-three weeks. Lindane 1% was used only after the four weeks of study period had passed and therefore, it did not influence the results in the least. Treatment failure and resistance were likely due to partial non-compliance and/or improper application technique as well as not allowing enough time to pass following application before rinsing. Other potential causes may have included resistance to scabicides, inadequate treatment of contacts, reinfection due to overcrowding, poor hygiene such as longer fingernails, excessive scratching, and environmental factors such as shared bedding. Likewise, not following certain protocols, such as washing articles of clothing as directed, as well as a high burden of mite infestation may also have been contributors to the persistence of scabies. Moreover, host factors such as variability in the immune responses, genetic factors, and individual differences in mite susceptibility may have been present, however, it is nearly impossible to rule out all such potential causes before enrollment of subjects or exclude such cases with 100% accuracy, although our exclusion criteria did try to minimize such factors as much as possible. Complete non-compliance was not seen in any case during our study.

The treatments were received with positive feedback from patients in both groups regarding their improvement in quality of life [4]. Moreover, none of the participants exhibited any signs of an allergic reaction immediately following topical application in any group. Although some individuals reported experiencing mild irritation as an adverse event, only a total of 44 patients expressed such symptoms; 20 and 24 patients from the ivermectin and permethrin groups, respectively. However, it is important to emphasize that this irritability was mild, improved over time and no significant adverse event or major hindrance in compliance was noted. Throughout this trial, none of the patients observed worsening of their condition, and even treatment failures including the resistant cases reported reduced symptoms overall, with a marked decrease in the number of their scabetic lesions compared to when the trial commenced. Although the study period lasted only four weeks, the remaining cases were closely followed, with them and their contacts receiving rigorous treatment. Ultimately, all the patients were treated successfully in both of the groups.

## Discussion

Multiple therapies have been proven to be effective in the treatment of scabies. Nonetheless, achieving successful outcomes often necessitates the active involvement of the entire family or community in order to prevent reinfection from taking place once again. One approach to mitigating itching triggered by scabies involves the use of antihistamines [19,20]. Scabies mites are microscopic parasites that burrow into the epidermis, breaking through the skin's protective barrier. This physical disruption of the skin's surface triggers an inflammatory response. This leads to itching due to the body's allergic reaction to the mites, their eggs, and their waste that continues until all the skin containing the dead mites is shed, which usually takes about two weeks. Continuing to have an itch does not indicate that the treatment isn't effective. It also doesn't mean that it needs to be repeated.

The advent of permethrin 5% skin lotion as an additional treatment option for scabies has been received with open arms due to its remarkable attributes: ease of application, elegant appearance, absence of unpleasant odor as well as non-staining properties on clothing. It must be emphasized that scabies can indeed induce skin irritation including itching, swelling, and redness which may experience temporary aggravation subsequent to permethrin treatment resulting from the absorption process of dead parasite proteins. Additionally, individuals might occasionally experience mild burning or stinging sensations [21]. Ivermectin is a proven and cost-effective option for treating scabies infections. While it is widely used to treat a number of parasitic infections, the US Food and Drug Administration (FDA) has still not approved it specifically for scabies treatment [22]. Safety with regard to pregnant and lactating women along with young children has yet to be proven, and further research is needed to establish its effects in these populations [22].

It should be noted that some patients did not experience the desired efficacy from using ivermectin, potentially due to its lack of ovicidal activity against parasites at early stages within the eggs when the parasites' nervous system has not fully developed [23,24]. This information however suggests that while generally effective against scabies parasites, there may be some limitations to its efficacy in certain cases [25]. Nonetheless, as the mites are all at different stages of their lifecycle, this does not result in a significant concern when opting to treat scabies using ivermectin. These elements could also account for the slight delay observed in full recovery from treatment with ivermectin. Since ivermectin has no known ovicidal effect, greater doses might be necessary to increase the cure rate [26,27]. Ivermectin 1% was administered to 69.3% of research participants, and according to Chhaiya et al., permethrin 5% lotion was used on participants, and after one week, 74.8% of them had a "complete cure" [14]. Ivermectin 1% lotion treatment resulted in a "complete cure" in 100.0% of participants, whereas permethrin 5% lotion treatment resulted in a "cure" in 99.0% of participants in follow-up after two weeks [14]. There was no significant difference in research groups after four weeks of follow-up to full recovery in the 210 participants studied (RR 1.02, 95% CI 0.96 to 1.08). According to research by Rao et al., 5% permethrin lotion was 81.3% effective at treating scabies [28]. In contrast to our findings with regards to scabies lesions, a study by Usha et al. found that more patients healed from scabies with the use of a single dose of permethrin versus a single dose of ivermectin [29]. The prolonged follow-up period may provide an explanation for this. The age of the subjects in a study by Ranjkesh et al. ranged from 4 to 72 years old [30]. Participants in the 5% permethrin group had a mean age of 42.56 with a standard deviation of ±16.56 years, whereas patients in the ivermectin group had a mean age of 46.76 with a standard deviation of ±14.45 years. There were an equal number of patients with mild, moderate, and severe illness in each treatment group at the beginning of the research. Following treatment, 24 patients (80%) in the permethrin group and 14 patients (46.6%) in the ivermectin group both displayed improvement within the first week. By the second week, symptoms had improved in 22 patients (73.3%) in the ivermectin group compared to 28 (93.3%) in the permethrin group [30]. This result is significant, but additional research involving more patients would help to enlighten physicians further. To control this infection more effectively, community-level trials, preferably double-blinded, comprising larger patient populations, are necessary to compare the effects of ivermectin and permethrin. Ivermectin was almost as effective as permethrin and although it is more affordable, either of them may be given to patients in both in-patient as well as out-patient settings [29]. They also have an equal rate of compliance and can be prescribed safely in scabies accompanied by secondary eczema and ulcers or when topical treatments like lindane and benzyl benzoate may lead to substantial cutaneous or systemic adverse effects, not to mention, compliance issues [19,20].

Limitations

Blinding in some patients was difficult due to the fact that they had used topical permethrin in the past and were somewhat able to recognize it by its characteristic odor. The number of such patients was few when compared to the study pool and, thus, did not have a significant impact on the final results. The majority of the patients did not report any issues with compliance as the odor dissipated within a couple of hours after application. However, possible variations, if any, such as subtle differences in the consistency of the two different lotions, cannot be ruled out. Although not many, the exact cause of treatment failure could not be determined in those who remained infested at the end of the study.

## Conclusions

Our study demonstrated equivalent efficacy between ivermectin 1% and permethrin 5% as topical therapies for the treatment of uncomplicated scabies, as evidenced by comparable results. The minimal and easily manageable side effects highlight the favorable safety profiles of both treatment modalities. Importantly, no statistically significant distinctions emerged between the two treatment groups, with patients exhibiting consistent compliance throughout the trial, hence affirming the confidence with which both ivermectin and permethrin can be employed in a clinical setting. Further studies in larger cohorts could provide additional insights, enlightening clinicians and offering further affirmation of the efficacy and safety of these topical treatments, especially for use in patients receiving polypharmacy, where potential drug interactions may be of concern.
